# Supplementary material for: From sequence to enzyme mechanism using multi-label machine learning
Source: BMC Bioinformatics. 2014 May 19;15:150. doi: 10.1186/1471-2105-15-150 (PMC4229970; doi:10.1186/1471-2105-15-150)
Supplement: Additional file 2 — Java code of ml2db. Additional file ml2db_code.tar.gz contains the Java source code to run the multi-label machine learning experiments and save the results to database. The code’s Javadoc is included. [file 1471-2105-15-150-S2.zip › additional file 2/ml2db/utils/doc/uk/ac/ed/inf/utils/database/Table.html]

Table


---


|  |  |  |
| --- | --- | --- |
| **Overview** \| **Package** \| **Class** \| **Use** \| **Tree** \| **Deprecated** \| **Index** \| **Help** | |  |
| **PREV CLASS**   **NEXT CLASS** | **FRAMES**    **NO FRAMES**     **All Classes** |  |
| SUMMARY: NESTED \| FIELD \| CONSTR \| METHOD | DETAIL: FIELD \| CONSTR \| METHOD |  |


---


## uk.ac.ed.inf.utils.database Class Table

```
java.lang.Object
  uk.ac.ed.inf.utils.database.Table
```

---

``` public class Table extends java.lang.Object ```

An SQL database table definition (table name + columns)

**Version:**
:   10 Jun 2010

**Author:**
:   Luna De Ferrari luna.deferrari-at-ed.ac.uk

---

**Constructor Summary**

`Table(java.lang.String tableName,
java.util.Vector<TableColumn> columns)`
  
          List of primary key fields (starting from 0)

`Table(java.lang.String tableName,
java.util.Vector<TableColumn> columns,
DbManager dbManager)`


| **Method Summary** | |
| --- | --- |
| `TableRow` | `createRow(java.lang.Object[] values, TableManager manager)` |
| `java.util.Vector<java.lang.String>` | `getColumnNames()`             Gets column names for the table |
| `java.util.Vector<TableColumn>` | `getColumns()`             Get the `TableColumn` list |
| `DbManager` | `getDbManager()` |
| `TableManager` | `getTableManager(DbManager manager)` |
| `java.lang.String` | `getTableName()`             Get the table name |
| `boolean` | `hasKey()`             Gets the "has primary key" status for the table |
| `void` | `initialise(java.lang.String tableName, java.util.Vector<TableColumn> columns)` |

**Methods inherited from class java.lang.Object**

`equals, getClass, hashCode, notify, notifyAll, toString, wait, wait, wait`

**Constructor Detail**

### Table

```
public Table(java.lang.String tableName,
             java.util.Vector<TableColumn> columns)
```

:   List of primary key fields (starting from 0)

---


### Table

```
public Table(java.lang.String tableName,
             java.util.Vector<TableColumn> columns,
             DbManager dbManager)
```


**Method Detail**

### createRow

```
public TableRow createRow(java.lang.Object[] values,
                          TableManager manager)
```

---


### getColumnNames

```
public java.util.Vector<java.lang.String> getColumnNames()
```

:   Gets column names for the table

    :   **Returns:**: the column names

---


### getColumns

```
public java.util.Vector<TableColumn> getColumns()
```

:   Get the `TableColumn` list

    :   **Returns:**: the `TableColumn` list

---


### getDbManager

```
public DbManager getDbManager()
```

---


### getTableManager

```
public TableManager getTableManager(DbManager manager)
```

---


### getTableName

```
public java.lang.String getTableName()
```

:   Get the table name

    :   **Returns:**: a string with the table name

---


### hasKey

```
public boolean hasKey()
```

:   Gets the "has primary key" status for the table

---


### initialise

```
public void initialise(java.lang.String tableName,
                       java.util.Vector<TableColumn> columns)
```


---


|  |  |  |
| --- | --- | --- |
| **Overview** \| **Package** \| **Class** \| **Use** \| **Tree** \| **Deprecated** \| **Index** \| **Help** | |  |
| **PREV CLASS**   **NEXT CLASS** | **FRAMES**    **NO FRAMES**     **All Classes** |  |
| SUMMARY: NESTED \| FIELD \| CONSTR \| METHOD | DETAIL: FIELD \| CONSTR \| METHOD |  |


---
